# Green synthesis of silver and iron nano composites using aqueous extract of *zanthoxylum armatum* seeds and their application for removal of acid black 234 dye

**DOI:** 10.3389/ftox.2024.1288783

**Published:** 2024-03-18

**Authors:** Nadia Bashir, Saba Gulzar, Salma Shad

**Affiliations:** ^1^ Department of Chemistry Hazara University Mansehra, Dhodial, Pakistan; ^2^ Department of Chemistry, Faculty of Physical and Applied Sciences, The University of Haripur, Haripur, Pakistan

**Keywords:** green synthesis, zanthoxylum armatum, timur, acid black dye, adsorption

## Abstract

Green nanotechnology has gained attraction in recent years due to the growing awareness of the environmental and health risks associated with traditional methods of nanomaterial synthesis. In the present study, nanocomposite (NCs) of silver and Iron were prepared using Zanthoxylum Armatum seeds aqueous extract which acts as a reducing, stabilizing, and capping agent. The synthesized NCs were characterized using UV/Vis Spectroscopy, powder X-ray diffraction (XRD), Scanning Electron Microscopy (SEM), and EDX. The UV/Vis spectroscopy analysis of the NCs revealed the presence of a surface plasmonic resonance band occurring at 420 nm. Examination of the NCs through SEM demonstrated that they exhibited a nearly spherical morphology, with an average particle diameter measuring 54.8 nm. The crystalline nature of these NCs was verified through X-ray diffraction (XRD), and the calculation of crystallite size using the Scherrer-Debye equation yielded a value of 12.6 nm. The adsorption ability of newly synthesized nanocomposites was investigated against Acid Black 234 Dye. The results showed that a 0.5 g of NCs dose at pH 4 removed 99.3% of 10 mg/L of Acid Black 234 Dye within 60 min. Based on the findings of this research, it can be inferred that the that Ag-Fe NCs synthesized from Zanthoxylum Armatum seeds aqueous extract hold significant potential for addressing environmental pollution caused by Acid Black 234 Dye. The NCs were used as adsorbent for the removal of Acid Black 234 dye from the wastewater sample and showed 98% removal of dye from the commercial sample within 60 min. In this context, the research highlights that the environmentally friendly synthesis of Ag-Fe nanocrystals (Ag-Fe NCs) using Zanthoxylum Armatum as a mediator offers an efficient and cost-effective solution for mitigating environmental pollution.

## 1 Introduction

Zanthoxylum armatum, also known as winged prickly ash, rattan pepper, or Timur, is a fascinating plant belonging to the family Rutaceae (Jie et al., 2023). An aromatic, deciduous, spiny shrub reaches a height of 3.5 m and is endemic from Pakistan across Southeast Asia and up to Korea and Japan. It is one of the primary sources of the renowned spice Sichuan pepper for its unique taste and aroma in Chinese cuisine (Cai et al., 2022). In addition to its culinary significance, this plant is deeply used in traditional medicine to treat a variety of conditions, including toothache, stomachache, and diarrhea (Cicchetti et al., 2017). Moreover, Zanthoxylum armatum plays a role in essential oil production, contributing to aromatherapy with benefits like pain relief and relaxation. Small white flowers, red or purple berries, and a citrusy aroma make this plant a versatile addition to ornamental gardens. Its wide-ranging applications underscore its cultural and botanical importance.

Zanthoxylum armatum acts as a reducing agent, as revealed in studies conducted by Jie et al. (2023). The plant is rich in composition compounds with reducing properties, including Gallic acid, Quercetin, Rutin, and Cinnamaldehyde. Gallic acid and Quercetin both polyphenolic compounds, along with Rutin, a glycoside, and cinnamaldehyde, an essential oil, collectively contribute to the plant’s reducing abilities. These compounds are known for their antioxidant, anti-inflammatory, and antimicrobial properties. The diverse reducing properties of Zanthoxylum armatum find practical applications in several fields, including nanoparticle synthesis, degradation of pollutants, and treatment of diseases. Verma et al. (2023) successfully synthesized the zinc oxide nanoparticles (ZnO NPs) using plant extract. These nanoparticles proved beneficial for applications including water purification and antibacterial therapy, showcasing the plant’s potential in innovative and sustainable technologies. Barkatullah et al. (2015) reported the presence of zinc, iron, lead, sodium, potassium, manganese and other elements in different parts of this plant.

Acid Black-Dye 234 (AB-234), a hazardous organic dye widely used in the textile industry, poses significant threats to both the environment and human health (Gonclaves et al., 2019; Hussain et al., 2021). This toxic dye, if ingested or inhaled, can lead to severe health problems. Therefore, the removal of AB-234 from wastewater is necessary before discharged into the environment (Anirudhan et al., 2017; Said et al., 2019; Mahmood et al., 2020; Sadia et al., 2023). The broader context of environmental pollution, specifically the water pollution crisis and scarcity of freshwater resources, demands urgent attention (Nanthamathee et al., 2021).

Dyes, owing to their wide applicability in different industries, contribute significantly to environmental degradation (Qin et al., 2020). An estimated 7 × 10^5^ to 1 × 10^6^ tons of dyes are produced annually and used in industries such as textiles, pulp, paper, plastics, food, cosmetics, and color photography (Reddy et al., 2020). With the widespread use and applications of dyes, an extensive proportion of dye-containing wastewater is disposed of and released into the environment without treatment (Fiorenza et al., 2020). Alarming disposal practices result in about 50% of dye utilization by the textile industry being discarded during the dyeing process, further contributing to environmental degradation (Zhang et al., 2020; Liang et al., 2021). Due to their permanence and high toxicity, dyes pose serious threats due to their toxicological, mutagenic, and carcinogenic properties (He et al., 2012; Kittapa et al., 2020; Nguyen et al., 2020).

To compact dye pollution, several approaches have been developed, such as filtration, ozonation, degradation, precipitation, coprecipitation, coagulation, high-performance liquid chromatography, sonophotocatalytic degradation and the use of polymeric adsorbents (Awual et al., 2019; Al- Musawi et al., 2022; Al- Hawary et al., 2023). Yilmaz et al. (2022) synthesized Fe_3_O_4_/ZnO/GO magnetic photocatalyst through low-temperature hydrothermal methods and applied it to remove acid blue 113 dye. Sillanpää et al. (2023) investigated the adsorption of Acid Orange 7 (AO7) by the Polypyrrole/nanosilica composite (PPN/SiO_2_), by exploring various parameters. The successful degradation of industrial azo reactive dyes, including Corafix Red ME4B, Corazol Golden Yellow, and Corazol Black BX, has been demonstrated by Sriram Krishnamoorthy et al. (2021). This achievement was realized through the activation of persulfate (S_2_O_8_^2−^) using Flyash (FA)-Fe_3_O_4_, FA-Fe_3_O_4_-Ag, and FA-Fe_3_O_4_-Ag-Cu magnetic composites as activators, showcasing their effectiveness in the dye degradation process. These methods play a crucial role in mitigating the adverse environmental health In the recent study, the Silver and Iron nanocomposites were synthesized by using the aqueous seed extract of zanthoxylum armatum as a reducing and stabilizing agent. The synthesized NCs were used as adsorbents for the removal of AB-234 dye from the real sample. The NCs were synthesized at optimum conditions and characterized before adsorption. Different parameters for the adsorption of dye were optimized and used for the removal of dye from wastewater samples.

## 2 Materials and methods

### 2.1 Reagents and apparatus

AB-234 dye (Sigma Aldrich), silver nitrate (Merck), and Ferrous sulfate (Merck) were purchased and used. Distilled water was used throughout the experiment. A hot plate, A REX—C410 - KOREA Model, was used as a heating mantle. For the filtration centrifuge, Centurion Scientific EB-Series, an orbital shaker, and an electric digital balance were used. A micropipette was used to calibrate the solvents in low amounts.

### 2.2 Collection and synthesis of aqueous extract of zanthoxylum armatum seeds

Fresh seeds of Zanthoxylum Armatum seeds were collected from the hills of Shinkiari (3445 feet above sea level) District Mansehra, Pakistan. It was washed with running tap water to remove the dust particles, rinsed with distilled water and air dried at room temperature for 2 h 30 g of washed and dried seeds were taken in 250 mL of deionized water and the mixture was boiled at 80°C for 30 min on a hot plate and filtered using Whattman filter paper (diameter: 12.5 cm). The extract was dark brown and this was used to synthesize NCs of silver and iron.

### 2.3 Preparations of standard solutions and synthesis of Ag-Fe nano composites

**FIGURE 1:**
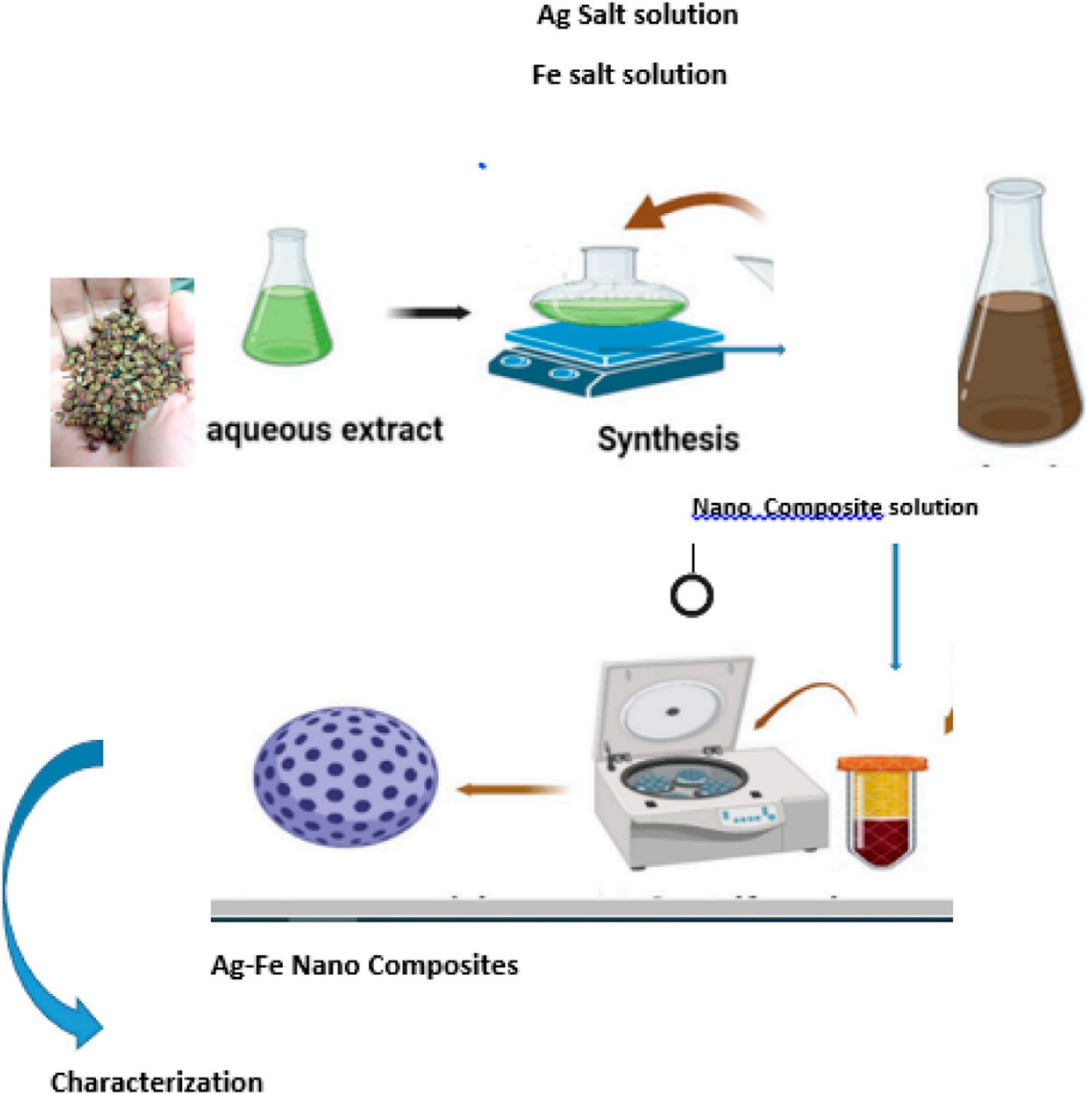
Graphical abstract.

An equimolar solution of 0.1 M silver nitrate and 0.1 M Iron sulfate was prepared by taking 1.7 g of silver nitrate and 1.52 g of Iron sulfate into 100 mL of distilled water in two separate volumetric flasks. Ag-Fe Nanocomposites were synthesized using a nontoxic and cost-effective approach, under controlled conditions following optimization of the synthesis parameters (Figure 1). The NCs were prepared using Extract, 0.1 M AgNO_3_ solution, and 0.1 M FeSO_4_ solution at a volume ratio of 1:1:4 respectively and heated at 90 °C on a hotplate for 15 min. The Nanocomposites appeared in the flask on heating. The flask was removed from heating and cooled to room temperature. The NCs were separated by centrifugation at 5500rpm for 15 min. The separated NCs were washed with distilled water and dried in an oven using a petri dish. The NCs were stored in sealed tubes and different characterizations were done for these NCs. The reduction of Fe and Ag ions was observed by carefully monitoring an aliquot (1 mL) at different time intervals (1–5 min) by UV-visible spectrophotometer using a lambda 25 (Perkin Elmer, Shelton, CT 06484, United States of America). A spectral scanning analysis between 200 and 800 nm was performed, and the absorption maximum for the synthesized NCs was found at 420 nm. The synthesized NCs were dark brown. By using the 1:1:4 of Extract, silver precursor salt, and Iron precursor salt respectively about 1.04 g of dried NCs were obtained. To attain greater stability, maximum yield, maximum production, and controlled size of NCs, different parameters including the mixing ratio of precursor salt, temperature, and time of heating were optimized.

### 2.4 Characterization techniques

The synthesized NCs were characterized by elemental analysis (EDX), scanning electron microscopy (SEM), and X-ray crystallography (XRD) before the removal of the acid dye, which affected the adsorption process. The size and morphology of the NCs were characterized using scanning electron microscopy (SEM), and XRD analysis was performed to find out the size and shape of the NCs.

### 2.5 Optimization of wavelength and a calibration curve of AB-234 dye

To assess the effectiveness of synthesized NCs in eliminating AB-234 dye, different experiments were carried out to investigate adsorption under different conditions, including variations in pH, adsorption duration, dye concentration, temperature, and the quantity of NCs used for adsorption. A standard solution (200 mg/L) of AB-234 dye (Figure 1)was prepared by adding 17.2 g of dye to 100 mL of methanol in a volumetric flask (100 mL). A standard solution of AB-234 dye was prepared to determine the optimum wavelength using a reagent blank. The optimum wavelength was found to be 480 nm; at this wavelength, a calibration curve was plotted with lower concentrations (1–25 mg/L) using a reagent blank. All measurements were performed by using a UV-visible spectrophotometer using a lambda 25 (Perkin Elmer, Shelton, CT 06484, United States of America). The working standard was prepared by applying a dilution formula to the stock solutions.

The adsorption efficiency of NCs was calculated using the following Eq. 1

$$\%\text{ Adsorption}=\frac{\left(C_{o}\;-\;C_{t}\right)}{C_{o}}\times 100$$ (1)

where C_o_, is the initial concentration of Acid black 234 dye, and C_t_, is the concentration of dye after adsorption time, t.

### 2.6 Effect of different parameters of adsorption of AB-234 dye on NCs adsorbent

Different parameters, such as pH, adsorbent dose, the concentration of adsorbate, and contact time, were studied.

#### 2.6.1 Effect of pH on adsorption of AB-234 dye

Britton–Robinson buffer was prepared to study the effect of pH on AB-234 dye. The pH (1–7) was adjusted by adding 0.5 M HCl and 0.5 M NaOH solution. 0.1 g of the synthesized NCs was added to each flask containing AB-234 dye (100 mg/L). To adjust the pH, 5 mL of buffer solution was added to the flasks, shaken for 10 min, and kept at room temperature. Filtration was performed and each filtrate was measured against a reagent blank (at a wavelength of 480 nm).

#### 2.6.2 Effect of adsorbent dose

The effect of the adsorbent on the adsorption of AB-234 dye by the prepared NCs was studied. Different amounts of NCs, ranging from 0.1 to 0.5 g, were added to 100 mg/L AB-234 dye. Furthermore, 5 mL solution of the optimized pH 4 solution was added to each flask and shaken for 5 min. Filtration was performed, and the filtrate was measured against a reagent blank. It was observed that on increasing the amount of NCs the % adsorption increases due to the availability of more active sites of adsorption. The time of adsorption is decreasing as we increase the dose of NCs.

#### 2.6.3 Effect of contact time

To determine the impact of contact time on the adsorption ability of the NCs, several time intervals (10–120 min) were studied. The optimized amount of NCs (0.3 g) and solution with a pH of 4 was added to a 100 mg/L solution of the adsorbate. The contact time between the NCs and the dye solution affects the adsorption capacity of the NCs adsorbent. Absorbance was measured and recorded.

#### 2.6.4 Effect of concentration of adsorbate

The effects of various concentrations of AB-234 dye on adsorption were studied. Various adsorbate solutions ranging from 0.5 to 70 mg/L were prepared by adding 5 mL of an optimized solution with a pH of 4 and 0.3 g of the optimized adsorbent. The solutions were shaken for 5 min, filtered, and measured against a reagent blank to record the results. All optimization steps were performed in triplicate. The method was found suitable for adsorption of low and high concentrations of dye.

#### 2.6.5 Regeneration of used NCs and determination of their reuse ability

0.3 g of used NCs were placed in a flask and 100 mL of distilled water was added to them. The NCs were then heated on a hot plate at 70°C for varying time intervals ranging from 10 to 120 min. After every 10 min, the absorbance of the solution was measured at 480 nm to determine the desorption of AB-234 dye and the time required to regenerate the NCs. The regenerated NCs were dried in oven for 2 h and Reused for the adsorption of 100 mg/L AB-234 dye using the same method.

#### 2.6.6 Application of the synthesized NCs for removal of AB-234 dye from commercial waste water sample

Water samples were collected from the textile industry to test for the presence of AB-234 dye. The initial concentration of the dye in the wastewater was determined using the external standard addition method and found to be 120 mg/L. To reduce the concentration of the dye, 0.3 gm of NCs were added to the wastewater solution at a pH of 4. The solution was shaken for 5 min and left to stand for 80 min to allow for maximum adsorption of the dye. The absorbance of the solution was measured at 480 nm and the concentration of the dye was calculated using the calibration curve of the standard AB-234 dye. The wastewater sample was analyzed in triplicate to ensure accurate results.

## 3 Results and discussion

### 3.1 Characterization of NCs using UV-visible spectrophotometry

**FIGURE 2:**
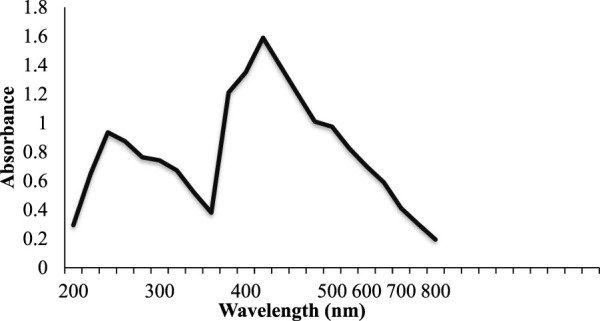
UV/Visible spectra of Ag-Fe NCs.

The green synthesis of the NCs was observed through UV visible double beam spectrophotometry, with a representative spectrum shown in Figure 2. The absorption peak was obtained at 420 nm with an absorbance (A) of 1.590. This is because the bandgap of Ag-Fe NCs is typically around 2.4 eV, which corresponds to a wavelength of light of about 500 nm (Wazu.K et al., 2008). Silver nanoparticles, in particular, are known for their strong surface plasmon resonance (SPR) properties. SPR in Ag-Fe NCs can lead to a sharp absorption peak in the visible range due to collective oscillations of electrons on the nanocrystal surface. SPR typically results in a sharp absorption peak in the visible range, often in the green to blue part of the spectrum. If the Ag-Fe NCs have a core-shell structure or contain silver nanoparticles on their surface, the presence of silver nanoparticles can contribute to an additional absorption peak related to SPR.

### 3.2 SEM-EDX characterization of biosynthesized NCs

**FIGURE 3:**
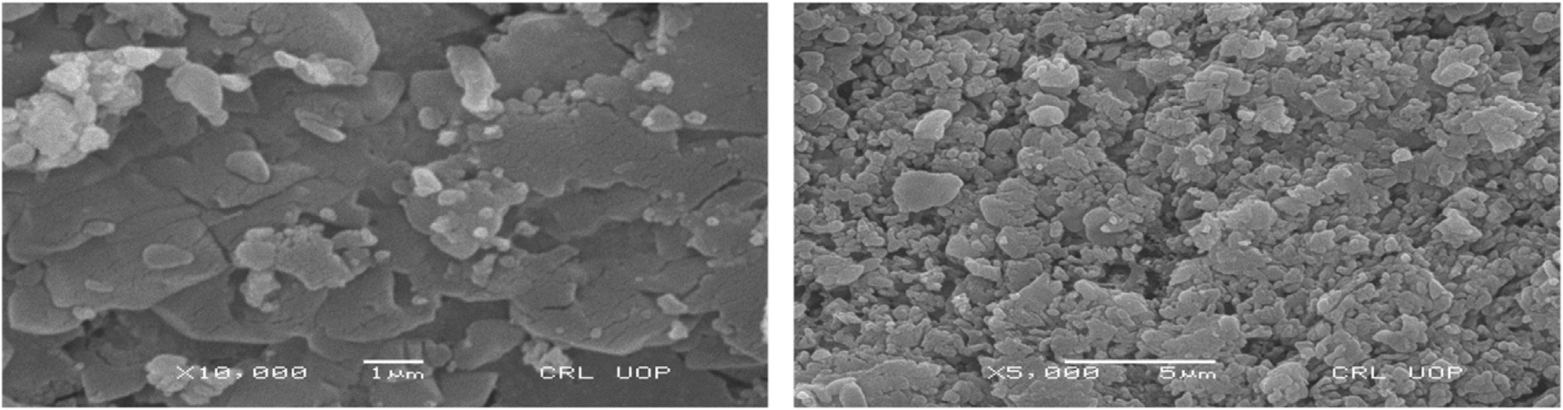
SEM image of Ag-Fe Nanocomposites.

**FIGURE 4:**
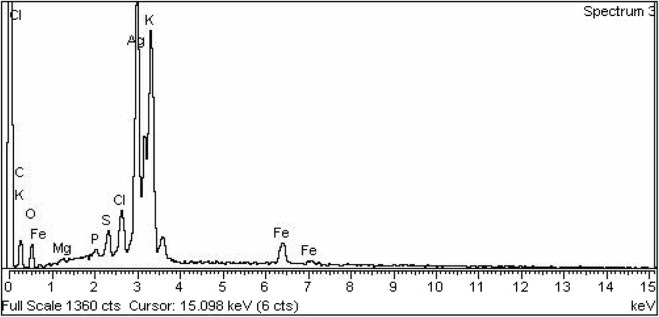
EDX spectrum of Ag-Fe Nanocomposites.

A representative SEM image and its corresponding EDX spectrum are shown in Figure 3 and Figure 4 respectively.

SEM examination was done to study the surface morphology of the nanocomposite made from seed extract, Ag, and Fe solutions. The shape of the nanocomposite was spherical or globular, with agglomeration. ImageJ software was used to determine the average particle size, which was found to be 54.8 nm.

EDX spectroscopy was used for the elemental analysis. The strong peak was observed for Ag with a % weight of 50.36, while the observed % weight of Fe and O were 5.94 and 15.46 respectively (Table 1). Table 1 displays the percentage of other elements phosphorous, sulfur, and magnesium indicating that these elements are from plant extract because plants absorb these from the soil. Based on the EDX study the proposed chemical formula of the nanocomposite is Ag_3_O_2_Fe.

**TABLE 1:** EDX analysis of Nano composites.

Element	Weight %	Atomic %
C K	5.90	18.68
O K	15.46	36.77
Mg K	0.32	0.50
P K	0.53	0.65
S K	1.82	2.17
Cl K	2.84	3.05
K K	16.84	16.38
Fe K	5.94	4.05
Ag L	50.36	17.76
Totals	100.00	100.00

The bold value represents the % result of all the elements present in the final product.

### 3.3 XRD analysis of silver and iron nanocomposites

**FIGURE 5:**
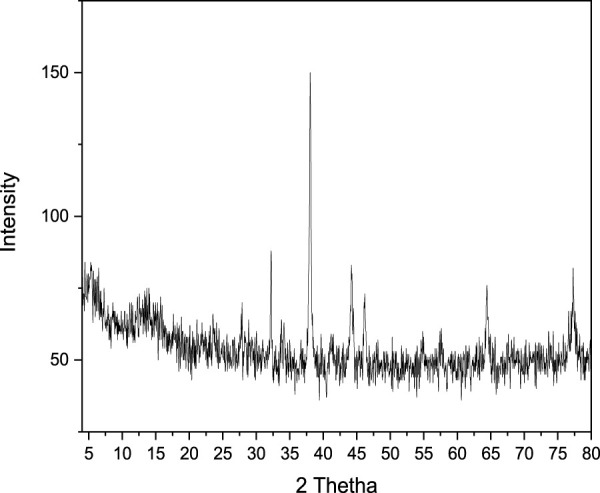
XRD spectrum of synthesized Nanocomposites.

The XRD pattern (Figure 5) indicates that the composite material was synthesized in the nano-scale range. The 2θ values were observed at 38, 32.5, 43, 46, 64.9, and 77.5. Based on the 2θ values the strongest peak at 38° corresponds to an interplanar spacing of 1.25117 Å. This is the (111) plane spacing of a face-centered cubic (fcc) material. The other peaks can also be indexed to the fcc crystal structure, but they are weaker, which suggests that the nanomaterial is not perfectly spherical. The most likely shape of the nanomaterial is a sphere or a cube with some surface defects. The average size of the nanomaterial was 12.6 nm, based on the Scherer, equation i.e.,

$$D=\frac{K\lambda }{\beta \mathrm{CoS}\theta }$$ (2)

### 3.4 Standardization of calibration curve

**FIGURE 6:**
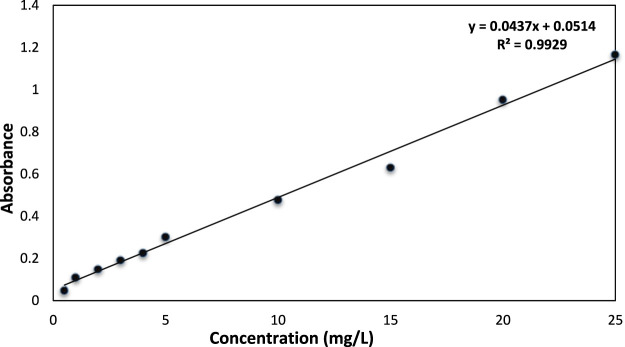
Calibration Curve determination at different concentrations of AB-234 dye.

The standard (200 mg/L) solution of AB-234 dye showed various working solution concentration ranges of 1–25 mg/L in the dilution formula (C_1_V_1_ = C_2_V_2_) (Patil et al., 2020). Absorbance was measured at λmax = 480 nm, which is the optimized wavelength for AB-234 dye, using a UV/Visible double-beam spectrophotometer. A linear curve of absorbance *versus* concentration (Figure 6) was obtained against the solvent blank (methanol), with a correlation coefficient (rˆ 2) of 0.992, indicating the suitability of this method for determining the concentration of AB-234 dye in solution.

#### 3.4.1 Effect of pH, adsorbent dose and contact time

**FIGURE 7:**
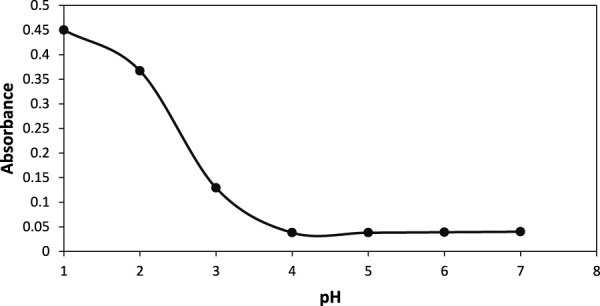
Effect of pH on adsorption of AB-234 dye.

**FIGURE 8:**
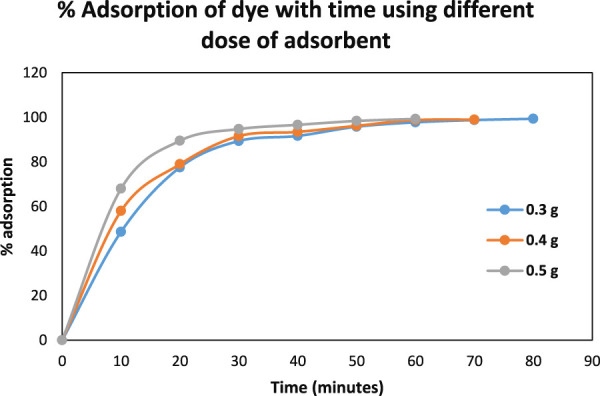
% adsorption of Dye with time using different doses of adsorbent.

The effect of the pH, adsorbent dose and contact time on the adsorption of AB-234 dye by the prepared NCs was studied. The effect of pH on adsorption ability of NCs was studied. It was observed that maxiumum adsorption was taking place at pH 4 Figure 7. Different amounts of NCs, ranging from 0.3 to 0.5 g, were added to 10 mg/L AB-234 dye. Furthermore, each flask was shaken for 5 min after adjusting the pH. Filtration was performed, and the filtrate was measured against a reagent blank. It was observed that on increasing the amount of NCs from 0.3g to 0.5 g the % adsorption increases from 48% to 68% within 10 min. This is due to the availability of more active sites of adsorption on increasing the dose of adsorbent. The effect of contact time was also observed from 10 min to 80 min. The adsorption of dye increases with time and reaches 99.39% within 80 min when 0.3 gm of NCs was used. While the time of adsorption was further reduced to 70 min and 60 min with an % adsorption of 99% and 99.3% when the amount of NCs was 0.4 g and 0.5 g respectively (Figure 8). The method was compared (Table 2) with different methods reported by researchers and found more efficient and economical.

**TABLE 2:** Comparison of present work with Literature value for the Removal of AB-234 dye.

System used	% Degradation	Time	References
CS-ZnS-NPs	96.7	100 min	Aziz,A et al. (2020)
MIPs	94	40 min	Sadia,M et al. (2022)
Iron oxide nanoparticles immobilized Aspergillus flavus manganese peroxidase	85–92	24 h	Kalsoom,U. et al. (2022)
Ag-Fe NCs	99.3	60 min	present work

#### 3.4.2 Regeneration and reuse potential of NCs in the degradation process

**FIGURE 9:**
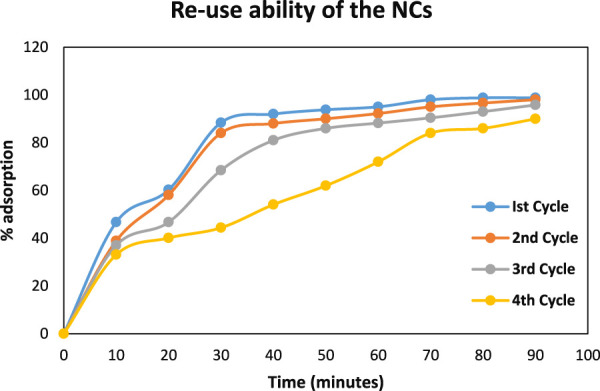
Reuse ability of Nanocomposites.

The potential for recyclability of NCs within the degradation process of AB-234 dye was investigated in four cycles for 120 min each. The NCs was regenerated on heating at 70°C for 90 min following each utilization. The NCs were separated by decantation, rinsed with distilled water, and then subjected to drying in an oven at 70°C for a duration of 2 h. The NCs was cooled at room temperature and then used in the degradation of a fresh solution of 100 mg/L AB-234 dye. The adsorption efficiency of NCs was monitored after each cycle, and the outcomes were graphically represented in Figure 9. The adsorption ability of synthesized NCs on AB-234 dye was observed to have a very small decline from 98.8% (first time) to 90% fourth time). The minor reduction in adsorption efficiency observed in each successive recycling cycle can be ascribed to the adsorption of dye molecules on the active sites of the NCs. These findings validate the NCs capacity to be reused as adsorbents for at least four cycles.

#### 3.4.3 Testing of Ag-Fe NCs on the commercial wastewater sample

The Synthesized NCs were further applied to samples obtained from the industry. It is important to have an idea about the dyes present in industrial wastewater samples because it is of utmost importance that the sample contains the specific dye that is going to be tested. Approximately 50 mL of the wastewater sample was taken from the industry after confirming the presence of the dye. To adjust the pH to 4, 5 mL of buffer was added to the solution. The NCs (0.3 g of NCs were added to the prepared solution and shaken for 5 min. The sample was maintained for 80 min and then filtered. The absorbance of the filtrate was measured and calculated. Each analysis was done in triplicate the % adsorption was calculated, and it was found that 98% of the dye was adsorbed from the solution by the NCs.

## 4 Conclusion

In conclusion, this study has detailed an economically viable and environmentally friendly approach for the green synthesis of Ag-Fe nanocomposites, employing aqueous extracts from Zanthoxylum armatum seeds as both reducing and capping agents. These newly synthesized nanocomposites underwent comprehensive characterization via UV/Vis spectroscopy, SEM, EDX, and XRD analytical techniques. The UV/Visible spectrum exhibited a distinctive SPR band at 420 nm, which was attributed to the presence of Ag-Fe nanocomposites. EDX analysis confirmed the composition of Ag, Fe, and O within the nanocomposites. SEM micrographs affirmed the spherical morphology of the nanocomposites (NCs), while XRD analysis established their crystalline nature, with a calculated crystallite size of 12 nm.The NCs were effectively employed as adsorbents for the removal of Acid Black 234 dye, displaying remarkable performance. At 480 nm, the % adsorption efficiency of the NCs reached 99% within a 60-min timeframe when 0.5 g of NCs were utilized.

The desorption studies were carried out to regenerate the used NCS and reused for the removal of acid dye. The NCs were regenerated on heating and successfully reused for the removal of acid dye with a removal efficiency of 98%. The synthesized NCs were also used for the removal of dye in real water samples. The results confirmed the successful use of newly synthesized NCs in environmental remediation of Acid Black 234 Dye.

## Data Availability

The raw data supporting the conclusion of this article will be made available by the authors, without undue reservation.
